# Patterns and outcomes of vascular access in hemodialysis: a nationwide registry-based study from Romania

**DOI:** 10.1080/0886022X.2025.2514830

**Published:** 2025-06-12

**Authors:** Gabriel Ștefan, Eugen Podgoreanu, Gabriel Mircescu

**Affiliations:** a Romanian Renal Registry, “Dr. Carol Davila” Teaching Hospital of Nephrology, Bucharest, Romania; b University of Medicine and Pharmacy "Carol Davila", Bucharest, Romania

**Keywords:** Hemodialysis, arteriovenous fistula, central venous catheter, survival analysis

## Abstract

**Background:**

Vascular access type significantly influences outcomes in hemodialysis (HD) patients. Arteriovenous fistulas (AVFs) are preferred due to lower morbidity and mortality, while central venous catheters (CVCs) are associated with higher risks. This study, based on national registry data, examines vascular access patterns and their impact on mortality in Romanian HD patients.

**Methods:**

A retrospective cohort analysis was conducted using Romanian Renal Registry data from patients initiating HD between 2020–2022, with follow-up until December 31, 2023. Vascular access was categorized as AVF, temporary CVC, or tunneled CVC. Survival analysis employed Kaplan–Meier curves and Cox proportional hazards models to evaluate the impact of vascular access type on mortality.

**Results:**

Among 6,863 incident HD patients, 20% initiated HD with an AVF, while 55% used temporary CVCs and 25% tunneled CVCs. AVF use increased to 53% in prevalent patients. Patients starting HD with AVFs had significantly better survival (39.1 months) compared to temporary (32.5 months) and tunneled CVCs (33.9 months). Mortality risk was over twofold higher in patients with CVCs at initiation. Transitioning from a CVC to an AVF significantly improved survival (HR 0.27, 95% CI 0.23–0.31).

**Conclusions:**

The high CVC use at HD initiation underscores the need for improved CKD diagnosis and pre-dialysis nephrology care in Romania. Early AVF placement or timely conversion from CVC to AVF significantly enhances survival. These findings support a patient-centered approach to vascular access, emphasizing tailored care and enhanced pre-dialysis planning to optimize outcomes.

## Introduction

The type of vascular access used in maintenance hemodialysis (HD) has a significant impact on patient morbidity and mortality [1–4]. Arteriovenous fistula (AVF) is generally recommended due to a lower rate of complications as compared to other options. In contrast, central venous catheter (CVC) use is linked to higher risks of infection, cardiovascular events, and to an overall higher mortality [4]. However, both vascular access choice and patient’s outcome seems also influenced by patient-related factors. Nonetheless, creation of a permanent vascular access, such as AVF or arteriovenous graft (AVG), and maintenance of its long-term functionality is critical to the reduction of CVC-associated risks [5].

The Dialysis Outcomes and Practice Patterns Study (DOPPS) analyzed data from 20 countries and reported an AVF use ranging from 19% to 84% in incident HD patients, and from 49% to 92% in prevalent HD patients [1]. Variations in AVF maturation times and patency rates were also globally reported [6].

The optimal timing for vascular access creation remains unclear. Delaying AVF creation increases the risks associated with starting dialysis using a CVC, whereas early AVF creation may be unnecessary if the patient never needs dialysis initiation [7,8].

On the other hand, the type of vascular access, both in incident and prevalent patients was used as an indicator of the quality of nephrology care. Some studies suggest that pre-dialysis AVF creation reduces sepsis-associated hospitalizations and mortality, while others found no benefit of early AVF creation on survival in the first year after HD initiation. Moreover, the advantages of AVF over CVC at HD initiation have been questioned, as not all patient-related confounding factors were adequately accounted for in survival analyses [9–12].

Currently, there are no registry-based, nationwide data from South-East Europe on vascular access. In 2020, it became mandatory to report to Romanian Renal Registry (RRR) the type of vascular access at each HD session. Consequently, our study aimed to describe the vascular access choices and to assess the impact of vascular access type on mortality in incident HD patients.

## Methods

### Data sources

Data were obtained from the Romanian Renal Registry database. As reporting to registry is compulsory, RRR collects data regarding over 97% of patient receiving kidney replacement therapy (KRT) by dialysis in Romania, a country with a population of 19,054,548 and 13,745 ESKD patients (721 per million population) on 31st of December 2023 [13].

### Study design

We conducted a retrospective nationwide cohort study in patients starting hemodialysis between January 1, 2020 and December 31, 2022, and followed to December 31, 2023, or until death, kidney transplantation and dialysis withdrawal.

The main point of interest was the effect of the type of vascular access on patients and outcome. We also examined relations between the type of vascular access and patients’ characteristics at baseline and at the end of study.

### Study participants

Only patients aged ≥18 years who initiated maintenance hemodialysis. Those who died, were transplanted or withdraw in the first 90 days of therapy, as well as those lost to follow-up were excluded.

### Exposure

The exposure of interest was the vascular access on day 1 of hemodialysis in patients who started dialysis between 2020 and 2022.

Additionally, we evaluated the effect of the switch from CVCs to AVFs, using as reference the type of vascular access in use at HD initiation and at the last HD session.

The vascular access was classified as AVF, AVG, temporary CVC and tunneled CVC.

### Covariates

The register collects data on patient age, sex, primary kidney disease, data on first KRT (date and type), history of KRT, and data on death (date and cause).

The primary renal diagnosis was summarized in six categories: glomerular diseases, diabetic kidney disease, vascular nephropathies (including arterial hypertension related nephropathy), tubulointerstitial nephropathies, other and not assessed.

### Outcome

The primary outcome was all-cause mortality during at least one-year follow-up period after hemodialysis initiation. The causes of death were summarized as cardiovascular, infectious, neurology, gastrointestinal, malignancy, other/not assessed.

### Statistical analysis

Categorical variables are presented as percentages, with comparisons performed using the χ^2^ test.

Continuous variables are displayed as means or medians with 95% confidence intervals (95% CI), depending on their distribution, and compared using either the Student’s *t*-test or the Kruskal–Wallis test, as appropriate.

Statistical analysis followed an intention-to-treat approach, where multiple switches between vascular accesses were disregarded. For incident HD patients, deaths recorded after the first 90 days were attributed to the type of vascular access used on day 1 of therapy.

Survival analyses were conducted using the Kaplan–Meier method, with comparisons made *via* the log-rank test. The intention-to-treat analysis employed the Cox proportional hazards (CPH) model to estimate hazard ratios (HR) and 95% CI for the three vascular access types (AVF, temporary CVC, tunneled CVC). The association between survival and variables was assessed using a multivariate CPH model.

A *p*-value of <0.05 was considered statistically significant.

## Results

### Vascular access at hemodialysis initiation

**Figure 1.**
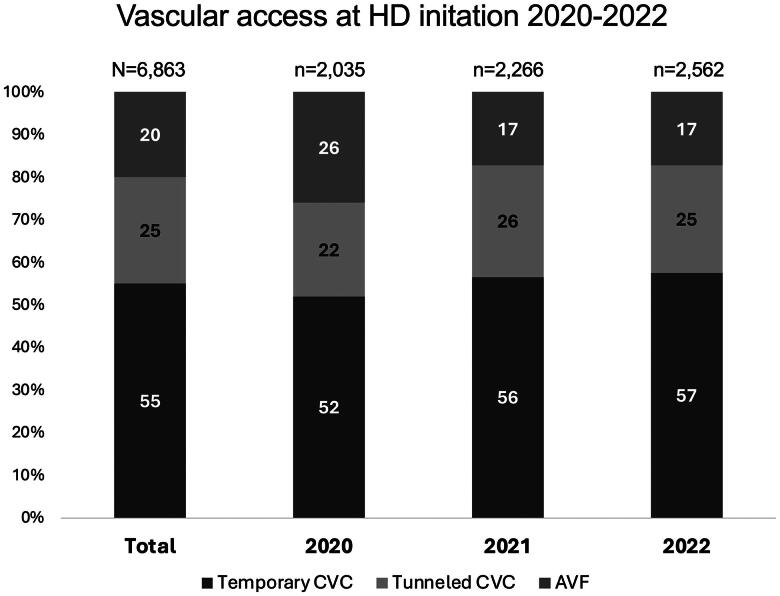
Vascular access at HD initiation 2020–2022. AVF, arteriovenous fistula; CVC, central venous catheter.

Among the 6,863 incident HD patients, 20% used AVF, 55% temporary CVC, and 25% tunneled CVC. This distribution was stable throughout the period of observation (Figure 1, Table 1).

**Table 1.** Patient characteristics at hemodialysis initiation and outcomes according to by vascular access type.

	All	AVF	Temporary CVC	Tunneled CVC	*p*
	*N* = 6,863	*n* = 1,355 (20%)	*n* = 3,793 (55%)	*n* = 1,715 (25%)	
Age, years	63 [52-70]	60 [50-68]	63 [53-71]	63 [51-70]	<0.001
Age groups, %					<0.001
75+ years	13	8	15	13	
55-75 years	55	54	56	55	
<55 years	32	38	29	32	
Male sex, %	61	60	62	58	0.06
Primary kidney disease, %					<0.001
Glomerular disease	14	14	14	14	
Diabetic kidney disease	12	9	14	9	
Vascular disease and HTA	8	5	9	5	
Tubulo-interstitial disease	10	10	11	10	
Other	4	3	3	2	
NA	52	58	50	59	
Mortality, n (%)	1,653 (24)	170 (13)	1,056 (28)	427 (25)	0.001
Cause of death, %					0.001
Cardiovascular	57	62	58	53	
Infectious	15	12	14	17	
Neurology	6	6	6	7	
Gastrointestinal	3	3	2	3	
Malignancy	5	5	4	8	
Other/NA	14	12	15	16	

AVF: arteriovenous fistula; CVC: central venous catheter; HTA: arterial hypertension; NA: not assessed.

The median age of the cohort was 63 years, with AVF patients being younger (median 60 years) compared to those with temporary or tunneled CVCs (both 63 years, *p* < 0.001). Only 8% of patients with AVF were aged over 75 years as compared to 38% in those under 55 years. The cohort showed a male predominance, with no significant differences in vascular access distribution (Table 1).

Primary kidney disease distribution was generally similar across vascular access types for glomerular and tubulointerstitial diseases. However, diabetic kidney disease and vascular diseases were more frequent among patients using temporary CVCs. Additionally, the primary kidney disease was unknown in a significant proportion of cases, which may reflect late referral to nephrology care (Table 1).

### Vascular access and patients’ survival

**Figure 2.**
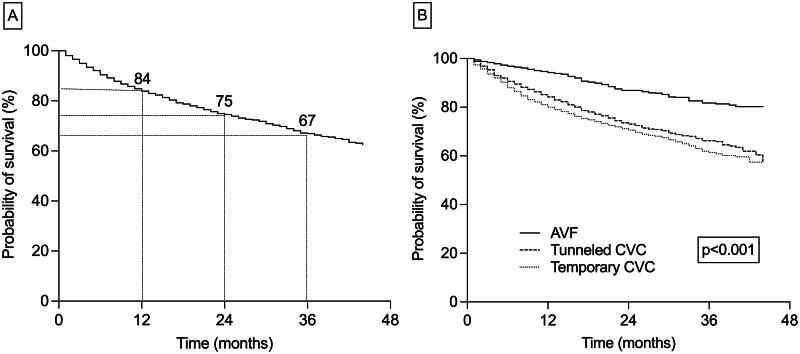
A. Cumulative probability of survival for the entire cohort; B. Survival comparison between different vascular access types at hemodialysis initiation (Kaplan–Meier analysis, log-rank test); AVF, arteriovenous fistula; CVC, central venous catheter.

During the observation period, 1,653 patients (24%) died. The mean patients survival for the entire cohort was 34.2 months (95% CI: 33.8–34.6). Survival rates at 12, 24, and 36 months were 84%, 75%, and 67%, respectively (Figure 2(A)).

Patients who started HD on AVF had a significantly higher mean survival time (39.1 (95%CI 38.5–39.8) months) as compared to those on temporary (32.5 (95%CI 31.9–33.1) months) and tunneled (33.9 (95%CI 33.1–34.7) months) CVCs. Mortality rates at 12, 24, and 36 months were also significantly lower in the AVF group (6%, 13%, 18%) than in both temporary (20%, 29%, 39%) and tunneled (16%, 27%, 34%) catheters users, respectively (Figure 2(B)).

Cardiovascular causes dominated across all groups, with higher rates in AVF users (62%), while infectious causes were more frequent in tunneled CVCs users (17%) (*p* = 0.001).

In the multivariate Cox regression analysis, age was a significant predictor of mortality, with a hazard ratio (HR) of 1.03 (95% CI: 1.03-1.04, *p* < 0.01), indicating a 3% increase in the risk of mortality per year of age. Female sex was associated with a 11% lower risk of mortality compared to males (Table 2).

**Table 2.** Risk factors for mortality in multivariate cox regression analysis.

	HR (95% CI)	*p*
Age, years	1.03 (1.03–1.04)	<0.01
Sex		
Male	Ref	
Female	0.89 (0.81–0.98)	0.02
Vascular access		
AVF	Ref	
CVC temporary	2.47 (2.09–2.90)	<0.01
CVC tunneled	2.18 (1.83–2.61)	<0.01
Primary kidney disease		
Diabetic kidney disease	Ref	
Glomerular disease	0.74 (0.61–0.89)	<0.01
Vascular disease and HTA	0.56 (0.45–0.69)	<0.01
Tubulo-interstitial disease	0.74 (0.61–0.90)	<0.01
Other	0.73 (0.62–0.89)	<0.01
NA	0.74 (0.63–0.84)	<0.01

AVF: arteriovenous fistula; CI: confidence interval; CVC: central venous catheter; HTA: arterial hypertension; HR: hazard ratio; NA: not assessed.

The type of vascular access at HD initiation significantly impacted mortality risk, independent of age, sex, and primary kidney disease. Patients using temporary or tunneled CVCs had a similar more than 2-folds higher risk of mortality risk (Table 2).

Regarding primary kidney disease, patients with diabetic kidney disease had the highest risk of death, while those with glomerular disease had a significantly lower risk. Similarly, patients with vascular disease, tubulo-interstitial disease, and other causes had a reduced mortality risk compared to diabetic kidney disease (Table 2).

### Vascular access change: transition from CVC to AVF

To evaluate the impact of the switch from CVCs to AVFs, we conducted a subanalysis focusing on patients who initiated HD with a CVC. We compared outcomes of patients who continued using a CVC and those who changed to an AVF. Of 5,316 patients analyzed (192 patients excluded due to missing data), 2,522 (48%) switched to an AVF, while 2,794 (52%) remained on a CVC.

Patients who changed to AVF were younger (median age 60 vs. 66 years, *p* < 0.001), more often male (66% vs. 56%, *p* < 0.001), and had a lower mortality (14% vs. 41%, *p* < 0.001). Cardiovascular diseases were the leading causes of death in both groups (Table 3).

**Table 3.** Comparison between patients who remained on CVCs and those who changed to AVFs.

	Change from CVC to AVF		*p*
	No	Yes	
	*N* = 2,794	*N* = 2,522	
Age, years	66 [56–73]	60 [49–68]	<0.001
Age groups, %			<0.001
75+ years	20	8	
55-75 years	57	54	
<55 years	23	38	
Male sex, %	56	66	<0.001
Primary kidney disease, %			<0.01
Glomerular disease	12	15	
Diabetic kidney disease	14	13	
Vascular disease and HTA	8	9	
Tubulo-interstitial disease	10	10	
Other	3	2	
NA	53	51	
Mortality, n (%)	1,136 (41)	340 (14)	<0.001
Cause of death, %			<0.001
Cardiovascular	57	56	
Infectious	15	13	
Neurology	5	7	
Gastrointestinal	3	3	
Malignancy	5	6	
Other/NA	9	11	

AVF: arteriovenous fistula; CVC: central venous catheter; HTA: arterial hypertension; NA: not assessed.

Patients who switched to an AVF had a significantly higher mean time of survival ((38.8 (95%CI 38.3–39.3) months) than those who continued using a CVC (27.1 (95%CI 26.3–27.8) months, *p* < 0.001). Moreover, Cox regression analysis showed that the switch from CVC to AVF significantly improved survival (HR 0.27, 95% CI 0.23–0.31, *p* < 0.001), independent of age, sex, and primary kidney disease.

## Discussion

In this national registry study from Romania, we examined vascular access choices and their impact on mortality in incident hemodialysis patients. To the best of our knowledge, this is the first registry study in South-East Europe to describe the vascular access patterns in hemodialysis. The use of AVF at HD initiation was low (20%) but increased in prevalent HD patients to 53%. However, only 48% of the patients who started on CVC switched to AVF during the study period. Patients who started HD with a CVC had an over 2-fold higher risk of all-cause mortality than those using a native AVF, while switching from CVC to AVF significantly improved survival.

### Vascular access at HD initiation

Ideally, patients needing KRT should make an informed choice between hemodialysis, peritoneal dialysis or kidney transplantation after thorough counseling. In those opting for HD, the choice of best access needs a careful evaluation. AVF is preferred in patients having an acceptable prognosis and suitable blood vessels. As AFV needs around 6 weeks to mature, pre-dialysis nephrology care is required for optimal timing of AVF creation [4]. Consequently, the CVC use varies depending on patients’ characteristics and availability and quality of pre-dialysis care.

Catheters use can also be imposed by the need of emergency HD initiation, resulting either from primary kidney disease (rapid decline in kidney function) or from late CKD diagnosis in primary care, followed by late referral to nephrologists. Also, deficiencies in specialized care (nephrology and vascular surgery) can play a role.

According to DOPPS data, the use of AVF at hemodialysis initiation varies significantly across countries, ranging from 84% in Japan to as low as 28% in the United States and Canada. European countries like Germany, Italy, and Spain report intermediate rates of AVF use, around 53–58% [1]. The rate observed in our study (20%) falls closer to the lower end of this spectrum.

The results from our population indicate that age plays a significant role in the choice of vascular access. The AVF patients tended to be younger than those using either temporary or tunneled CVCs (median age 60 vs. 63 years). Patients under 55 years were more likely to have an AVF as those over 75 (8%), suggesting that younger patients may have either a better vascular suitability and lower comorbidities or higher opportunity for planned dialysis initiation, favoring AFV creation.

While the distribution of other primary kidney diseases was similar across access types, diabetic kidney disease was more common among patients using temporary CVCs, reflecting the complex health profiles and potential need of emergency HD initiation in patients with diabetes mellitus.

On the other hand, the primary kidney disease was unknown in a larger proportion than reported by ERA Registry (52% vs. 27%), and more than half of these patients used CVCs at dialysis initiation [14]. This suggests emergency HD initiation and suboptimal preparation for dialysis initiation due to late referral to nephrologists. Moreover, in many studies and in a metanalysis, late referral was linked both to a low AVF use and a higher mortality.

Thus, the high rate of CVC usage at HD initiation in our cohort seems to be due to a combination of patients’ characteristics with late nephrology referral. These underlines the need of programs addressed to both CKD diagnosis in primary care and monitoring in nephrological care.

While our study highlights important regional disparities in vascular access use, these findings offer valuable insights into the challenges faced by healthcare systems in South-East Europe. The high reliance on central venous catheters at hemodialysis initiation underscores gaps in early chronic kidney disease diagnosis and referral practices. By shedding light on these regional patterns, our study contributes to the broader understanding of how healthcare infrastructure, patient characteristics, and access to specialized care influence vascular access outcomes, thereby informing targeted interventions for improved patient management.

Moreover, these results are in line with previous reports and suggest the need for a tailored, patient-centered approach when choosing the type of vascular access at the right time, considering patient underlying health conditions, according to ‘the right access for the right patient at the right time’ concept) [6,15].

### Vascular access and patients’ survival

The patient’s survival was related to the type of vascular access. Patients using AVF at HD initiation had better survival rates than those using temporary or tunneled CVCs, starting from first year and continuing till the third year of observation. Our findings are consistent with studies from the U.S., Asia, Australia, New Zealand, and Europe, and metanalyses, all showing higher mortality risk in incident HD patients using catheters compared to those with AVF [16–23].

A nationwide cohort study in Japan demonstrated that creating permanent vascular access, such as an AVF, at least one month before hemodialysis significantly reduced all-cause mortality, with even greater benefits observed when access was established four months prior, underscoring the need of early intervention [22]. Moreover, a French registry study of 53,092 incident HD patients found higher mortality rates in those starting with a nonfunctional AVF access compared to those with functional access [12].

In our study, use of catheters at HD initiation was independently associated with a 2-fold higher risk of mortality. However, patient related factors, e.g. increasing age, male sex and diabetic kidney disease, were also independent predictors of mortality. Otherwise said, not only vascular access matters for patients’ survival, but also some characteristics of patients.

This is in line with other studies pointing to the importance of patients-associated factors, which can confound the benefits of AVF at HD initiation on outcome. A Canadian cohort study in incident HD patients found that predialysis AVF creation was associated with lower all-cause mortality in patients under 65 years. However, in those over 65 years, although the mortality risk was lower in the first two years after HD initiation, it increased thereafter. Moreover, only 2.3% of deaths were vascular access related. The authors concluded that the excess mortality in those treated by catheters seems to result from residual confounding, unmeasured comorbidity, or to treatment selection bias [10].

These authors’ conclusion is supported by an US study showing a mortality benefit with AVF (HR 0.50), yet even those with failed fistula attempts who later used catheters had a lower mortality than catheter-only treated patients (HR 0.66), suggesting that patient factors largely explain the differences [11].

Thus, our data argument that patient-related factors, not only the type of vascular access at hemodialysis initiation, are important for patient outcome, supporting KDOQI 2019 Guidelines for Vascular Access which stress the need for including demographic factors, prognosis, and blood vessels status when planning the vascular access [4].

### Vascular access change: transition from CVC to AVF

The switch from a CVC to an AVF significantly reduced mortality among patients who initiated HD with a catheter. This improvement persisted after adjusting for age, sex, and primary kidney disease, underscoring the critical role of AVF in enhancing survival. Similarly, a large cohort study of 78,871 patients found that starting HD with a CVC resulted in the highest mortality risk (HR 1.55, 95% CI 1.38–1.74), while early conversion to AVF within six months significantly improved survival (HR 1.04, 95% CI 0.97–1.13) compared to delayed conversion or remaining on a CVC [24]. Also, data from the previously cited French registry study support the utility of a timely change from CVC to AVF: patients switching from CVC to AVV had comparable survival rates with those using AVF from HD initiation [12].

These findings emphasize the importance of timely AVF placement and suggest that early conversion strategies should be prioritized to improve patient outcomes.

### Limitations

Our study has several limitations. First, its nonrandomized, retrospective design inherently limits the ability to infer causality. Although we adjusted for some confounding factors, unmeasured variables may still influence the observed associations. Second, the registry used does not collect data on comorbidities or laboratory values, which are known to significantly impact both mortality and vascular access choice; the absence of this information may affect the interpretation of our findings by introducing unmeasured confounding factors. Third, we applied an intention-to-treat censoring strategy rather than an as-treated approach, which may not fully capture changes in vascular access over time. Additionally, we lacked direct data on referral timing, with conclusions regarding late referral based on indirect indicators, such as the high proportion of unknown primary kidney disease and the elevated use of CVCs at dialysis initiation. Despite these limitations, the reliability of our findings is supported by the large cohort size and the robustness of data derived from a nationwide compulsory reporting system for incident HD patients.

## Conclusions

This national registry study from Romania reveals a high use of central venous catheters at HD initiation (80%), highlighting the need for improved CKD diagnosis in primary medical care and in pre-dialysis nephrology care. Our findings align with international data linking the CVC use to a higher mortality, emphasizing the benefits of HD initiation with an arteriovenous fistula or of a change from CVC to AVF. Additionally, the study underscores the significant influence of patient-related factors on both vascular access choice and catheter-related survival. These results support a patient-centered approach to vascular access, as recommended by recent guidelines, with a focus on enhanced CKD diagnosis in primary care and pre-dialysis nephrology care.

## Data Availability

The data underlying this article will be shared on reasonable request to the corresponding author.
